# To: Critical COVID-19 and neurological dysfunction - a direct comparative analysis between SARS-CoV-2 and other infectious pathogens

**DOI:** 10.5935/2965-2774.20230104-en

**Published:** 2023

**Authors:** Josef Finsterer, Fulvio Alexandre Scorza

**Affiliations:** 1 Neurology and Neurophysiology Center - Vienna, Austria; 2 Disciplina de Neurociência, Escola Paulista de Medicina, Universidade Federal de São Paulo - São Paulo (SP), Brazil

## To the Editor

With interest, we read the article by Teixeira-Vaz et al. on a prospective, single-center cohort study among 27 coronavirus disease 2019 (COVID-19) patients requiring mechanical ventilation > 48 hours for acute respiratory distress syndrome (ARDS).^(1)^ Compared to a disease control cohort, COVID-19 ARDS patients had an increased risk of neurological complications and corticospinal tract dysfunction.^(1)^ It was concluded that patients with severe COVID-19 should be systematically examined neurologically.^(1)^ The study is excellent but has limitations that are a cause for concern and should be discussed.

The main limitation of the study is that the clinical neurological examination was incomplete.^(1)^ The exam included only assessment of the sedation level, excitability of tendon reflexes, and presence/absence of Babinski’s sign.^(1)^ By evaluating only these three points, it is impossible to decide whether an individual patient has developed central nervous system or peripheral nervous system complications. A comprehensive clinical neurological examination is required to assess whether COVID-19 is complicated by neurological impairment. If the clinical examination indicates neurological abnormalities, instrumental investigations, such as cerebral imaging, electrophysiology, and cerebrospinal fluid studies, need to be initiated. Regarding the clinical exam, it is not clear why only the cortico-spinal tract functions were assessed. SARS-CoV-2 infections affect not only motor functions but also the entire body, particularly the entire central nervous system and peripheral nervous system.

A negative nasal or pharyngeal swab test using RT-PCR for severe acute respiratory syndrome coronavirus 2 (SARS-CoV-2) does not rule out that ARDS is due to COVID-19.^(2)^ COVID-19 patients can become superinfected due to immunodeficiency induced by SARS-CoV-2.^(3)^ We should know how many of the included patients were suspected of having not only SARS-CoV-2 infections but also other types of infections.

We also disagree with the definition of a seizure.^(1)^ Seizures are not necessarily associated with altered consciousness. Particularly, in focal seizures, consciousness may be intact. We also disagree with the definition of peripheral neuropathy. This term includes not only mononeuropathy, multiplex neuropathy, and polyneuropathy but also polyradiculitis, plexopathy, and small fiber neuropathy. Since the intensive care unit stay can be complicated by critical illness neuropathy or myopathy, we should know how many of the enrolled patients had neuropathy that was due to critical illness and not to SARS-CoV-2 infection.

A cerebrovascular disease not included in the evaluation is venous sinus thrombosis. Venous sinus thrombosis is commonly complicated by ischemic stroke and intracerebral bleeding. Venous sinus thrombosis has been repeatedly reported as a complication of SARS-CoV-2 infections.^(4)^ We should know how many of the included patients had a stroke due to venous sinus thrombosis.

We disagree that stroke, encephalitis, epilepsy, myelitis, and neuropathy can be diagnosed solely by means of the clinical exam. Diagnosing neurological conditions usually requires instrumental examinations to confirm or exclude a suspected diagnosis.

Overall, the interesting study has limitations that call the results and their interpretation into question. Addressing these issues would strengthen the conclusions and could improve the status of the study. COVID-19 patients with indications for neurological complications require comprehensive clinical and instrumental investigations to make an accurate diagnosis and initiate early and appropriate treatment.
